# Fourier Transform Ion Cyclotron Resonance Mass Spectrometry Applications for Metabolomics

**DOI:** 10.3390/biomedicines12081786

**Published:** 2024-08-06

**Authors:** Darcy Cochran, Robert Powers

**Affiliations:** 1 Department of Chemistry, University of Nebraska-Lincoln, 722 Hamilton Hall, Lincoln, NE 68588-0304, USA; darcy.lin@huskers.unl.edu; 2 Nebraska Center for Integrated Biomolecular Communication, University of Nebraska-Lincoln, Lincoln, NE 68588-0304, USA

**Keywords:** mass spectrometry, metabolomics, FT-ICR-MS, imaging

## Abstract

Metabolomics is an interdisciplinary field that aims to study all metabolites < 1500 Da that are ubiquitously found within all organisms. Metabolomics is experiencing exponential growth and commonly relies on high-resolution mass spectrometry (HRMS). Fourier transform ion cyclotron resonance mass spectrometry (FT-ICR-MS) is a form of HRMS that is particularly well suited for metabolomics research due to its exceptionally high resolution (10^5^–10^6^) and sensitivity with a mass accuracy in parts per billion (ppb). In this regard, FT-ICR-MS can provide valuable insights into the metabolomics analysis of complex biological systems due to unique capabilities such as the easy separation of isobaric and isomeric species, isotopic fine structure analysis, spatial resolution of metabolites in cells and tissues, and a high confidence (<1 ppm mass error) in metabolite identification. Alternatively, the large and complex data sets, long acquisition times, high cost, and limited access mainly through national mass spectrometry facilities may impede the routine adoption of FT-ICR-MS by metabolomics researchers. This review examines recent applications of FT-ICR-MS metabolomics in the search for clinical and non-human biomarkers; for the analysis of food, beverage, and environmental samples; and for the high-resolution imaging of tissues and other biological samples. We provide recent examples of metabolomics studies that highlight the advantages of FT-ICR-MS for the detailed and reliable characterization of the metabolome. Additionally, we offer some practical considerations for implementing FT-ICR-MS into a research program by providing a list of FT-ICR-MS facilities and by identifying different high-throughput interfaces, varieties of sample types, analysis methods (e.g., van Krevelen diagrams, Kendrick mass defect plot, etc.), and sample preparation and handling protocols used in FT-ICR-MS experiments. Overall, FT-ICR-MS holds great promise as a vital research tool for advancing metabolomics investigations.

## 1. Introduction

The field of systems biology combines many different ‘-omics’ techniques such as metabolomics, proteomics, lipidomics, transcriptomics, and genomics to better understand complex biological systems in a holistic manner by taking into account the interactions between the individual parts of the system [1]. Metabolomics is a recently developed omics technique that has become integral to understanding complex biochemical processes in both plant and animal models [2]. The metabolome consists of all the small molecules (<1500 Da) found within a cell, tissue, organ, or organism. In the current version of the Human Metabolome Database (HMDB, https://hmdb.ca/, accessed 1 August 2024), there are 248,097 metabolite entries, with more being added regularly [3]. Metabolism is often probed via analytical techniques such as one- or two-dimensional Nuclear Magnetic Resonance (NMR) Spectroscopy or Mass Spectrometry (MS) coupled to various separation techniques [4]. When combined, these two analytical techniques provide complimentary results that allow for a complete and accurate depiction of the metabolome. NMR can offer detailed information on molecular structures and chemical flux as well as precise quantitation in a non-destructive manner [5]. MS is more easily coupled to chromatographic separation techniques than NMR and has a higher sensitivity and specificity. As a result, MS can readily detect low-abundant fM compounds compared to the μM limits for NMR [6,7]. In particular, Fourier Transform Ion Cyclotron Resonance Mass Spectrometry (FT-ICR-MS) has uniquely unparalleled sensitivity and resolving power. ICR cells currently offer the highest-resolution mass accuracy, although quadrupole time of flight (QToF) and orbitrap analyzers are more commonly used in metabolomics studies since these mass spectrometers have faster scan speeds, are readily available, and are user-friendly [8].

FT-ICR-MS has already been proven to be a valuable asset to the field of metabolomics [8,9,10,11]. This review aims to build upon the existing literature, as well as to (1) provide a brief survey of FT-ICR-MS metabolomics research from the past decade (Table S1) and (2) offer some practical considerations for readers who may be interested in applying FT-ICR-MS to their own research.

## 2. FT-ICR-MS Fundamentals

**Figure 1:**
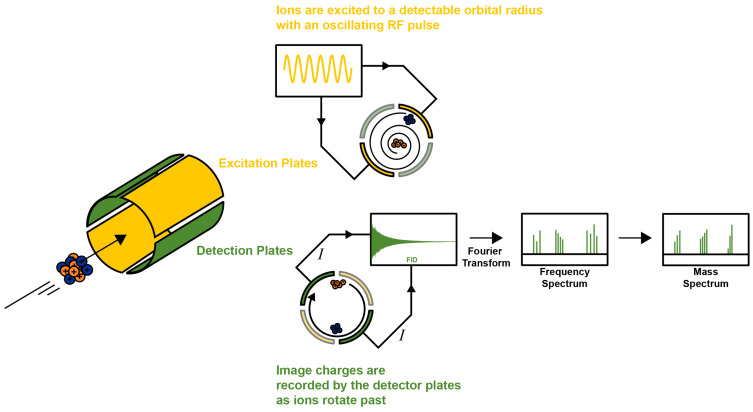
FT-ICR MS schematic. Graphic representation of Fourier Transform Ion Cyclotron Resonance Mass Spectrometry. The diagram illustrates the key operational principles of the FT-ICR-MS system, such as ion excitation to cyclotron motion, detection of image charges (*I*), and the subsequent transformation of the frequency spectrum into a mass spectrum.

Mass spectrometry is one of the primary forms of instrumentation used to characterize compounds across a wide range of fields and applications. It is sensitive, quantitative, widely accessible, and highly adaptable, with multiple mass analyzers to choose from when selecting an experiment [12]. The FT-ICR mass analyzer is a penning trap that traps ions in an electric and magnetic field. A radio-frequency pulse is then applied to the four plates in the ICR cell, causing ions to rotate in a synchronous cyclotron motion (Figure 1), which is directly correlated to the ion’s mass-to-charge ratio (*m*/*z*) [13]. A mass spectrum is generated by recording an ion’s frequency as it rotates within the ICR cell, which produces a free induction decay (FID) signal. The time-domain FID is then Fourier transformed into the frequency domain to produce peaks at the *m*/*z* of the ions [13]. Commercially available instruments have 7 T, 12 T, or 15 T magnetic field strengths, although 3 T–21 T homebuilt instruments are used in some research labs. Resolving power increases linearly with magnetic field strength. The stronger the magnetic field, the more stable the ion’s cyclotron frequency (Equation (1)), which offers higher resolving power as defined by Equation (2) [14].

(1) $$\omega_{c}=\frac{qB_{0}}{m}$$

(2) $$R=-\frac{qB_{0}}{m∆\omega_{50\%}}$$

where *ω_c_* is an ion’s cyclotron frequency in a magnetic field, *q* is the charge of the ion, *B* is the magnetic field strength, *m* is the mass of the ion, and *R* is the resolving power.

Further details on this process can be found in comprehensive reviews and textbooks on FT-ICR-MS [14,15]. The high resolving power and mass accuracy are the primary benefits of FT-ICR-MS over other MS platforms. FT-ICR-MS is unmatched, with a resolving power in the 10^5^–10^6^ range and a mass accuracy in parts per billion (ppb) [16,17,18], which allows for easier separation of isobaric and isomeric species. Thus, FT-ICR-MS facilitates a simpler analysis of complex samples and mixtures. Table 1 provides a summary of common MS analyzers and a few major figures of merit.

The ability to provide isotopic fine structure analysis (IFS) is another unique strength of FT-ICR-MS. Most elements are not monoisotopic and have stable, heavy isotopes such as ^2^H, ^13^C, ^15^N, ^17^O, ^18^O, ^18^O, ^33^S, ^34^S, and ^36^S. IFS is the fine-scale splitting of spectral lines due to the presence of isotopes of the constituent elements contributing to the *m*/*z* peak, but can only be observed when the mass spectrometer has sufficient sensitivity and resolving power [19]. The ratios of the heavy isotopes present in a single molecular ion create a unique pattern of peaks that comes from the isotopic contributions. Thus, IFS analysis provides the number of atoms present in the unknown compounds of interest. For example, alanine (C_3_H_5_NO) has a monoisotopic mass of 713.0371 amu and 28 possible isotopic permutations. A high-resolution mass spectrum of alanine would show peaks corresponding to the monoisotopic mass of [M]—71.0371 amu, [M + 1]—^13^C (72.0405 amu), ^15^N (72.0342 amu), ^2^H (72.0434 amu), ^17^O (72.0371 amu) and [M + 2]—^16^O (73.0414 amu). These masses only account for single isotopes and do not include charge carriers or other adducts in the calculations, although with high enough resolving power, peaks corresponding to ^13^C^12^C_2_^2^HH_4_^14^N^16^O, ^13^C^12^C_2_H_5_^15^N^16^O, or other permutations may be seen in the [M], [M + 1] or [M + 2] peak clusters. The intensity of these peaks is directly correlated to the natural abundance of the heavy isotopes and provides information on the number of atoms present in the compound of interest. The capability to accurately identify and quantify the constituent elements in compounds is extremely valuable, and this advanced molecular identification can be applied across various scientific fields [19].

## 3. FT-ICR-MS in the Search for Clinical Biomarkers

FT-ICR-MS brings unique capabilities that improve and facilitate the search for molecular biomarkers to diagnose human diseases and to assist with personalized medicine. Clinical biomarkers comprise a broad range of compounds, such as antibodies, metabolites, proteins, RNA, and other biomolecules, that can be measured accurately and reproducibly to indicate the presence of a pathogenic process or pharmacologic response [20]. A clinical biomarker may also provide a therapeutic target for a disease of interest [21]. For example, the proteins Aβ, α-synuclein, Tau, and TAR-DNA-binding protein 43 are known biomarkers for neurodegenerative diseases and are also important drug discovery targets [22]. The identification of a clinical biomarker is the desired goal of many omics studies, including metabolomics and lipidomics. Thus, changes in small molecules like lipids, amino acids, and energy metabolites may also signal the onset of a human disease such as neurodegenerative decline [22,23]. In this regard, the ability of FT-ICR-MS to provide high-level molecular identification is invaluable for the discovery of metabolite biomarkers. For example, FT-ICR-MS has been leveraged to study Alzheimer’s disease (AD) and identify potential clinical biomarkers. AD is associated with the accumulation of the amyloid-beta peptide in the medial temporal lobe and neocortical structures and is a debilitating neurodegenerative disease that is diagnosed in 500,000 adults annually. According to the 2023 Alzheimer’s Association annual report, there are 6.7 million Americans living with AD, with this number expected to double by 2060 [24].

**Figure 2:**
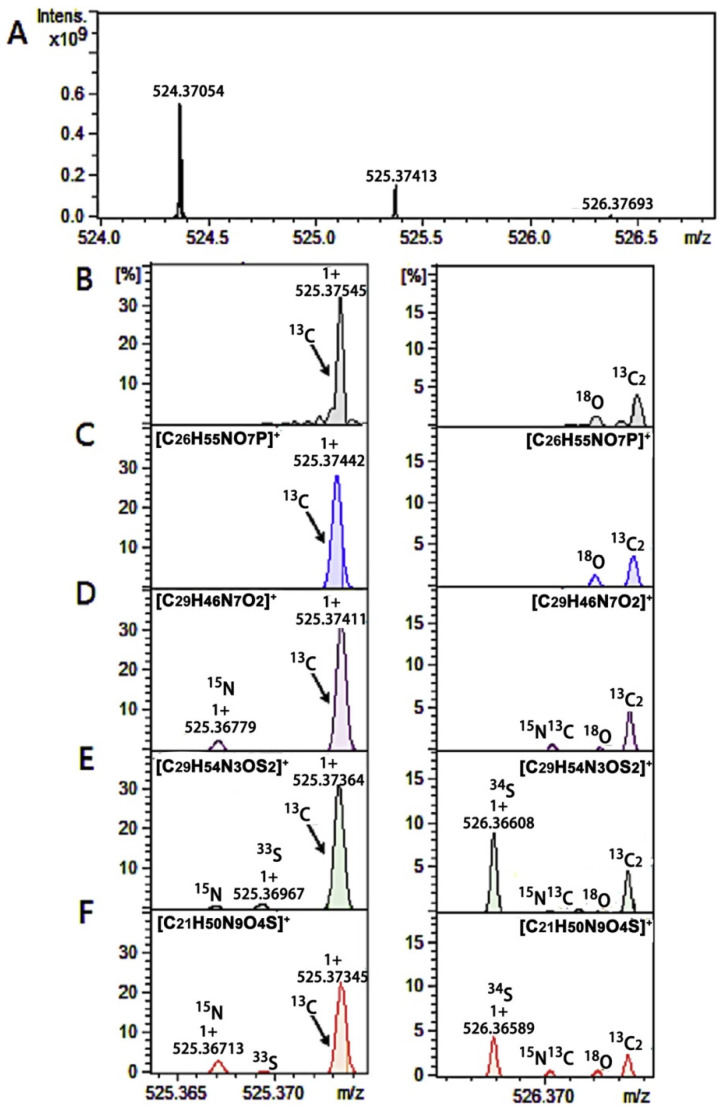
Example of fine isotopic structure analysis. Comparison of the experimental and the theoretical IFSs. High-resolution mass spectrometry of *m*/*z* 524.37054 (**A**). The experimental IFS of the ions with monoisotopic peaks at *m*/*z* 524.37054 (**B**) and four theoretical IFSs for [C_26_H_55_NO_7_P]^+^ (**C**), [C_29_H_46_N_7_O_2_]^+^ (**D**), [C_29_H_54_N_3_OS_2_]^+^ (**E**), and [C_21_H_50_N_9_O_4_S]^+^ (**F**). From the comparison between the experimental IFS and the theoretical IFSs, the ionic formula of the compound at *m*/*z* 524.37054 was assigned to [C_26_H_55_NO_7_P]^+^, the molecular formula of (**C**). Reproduced with permission from Elsevier [25].

Zhang et al. (2019) completed a serum metabolomics study of an AD rat model receiving an *Rhodiola crenulate* (RCE) treatment [25], which was previously shown to have therapeutic effects in AD patients. The goal of their study was to further elucidate the RCE-induced mechanism of restorative action [26,27,28]. A Bruker Solarix 7.0 Tesla FT-ICR-MS system was used to analyze the serum from AD rats treated with RCE. A total of 20 metabolites were identified that correlated with the progression of AD, and 17 of these metabolites were restored to control-like levels after RCE treatment. A unique two-stage FT-ICR-MS methodology was implemented to accurately identify these changing metabolites by using both high-performance liquid chromatography (HPLC)-FT-ICR-MS and direct injection (DI)-FT-ICR-MS. The first stage of the study yielded a standard LC-MS dataset that is commonly used in metabolomics, as well as sample fractions. Statistical analysis of the LC-MS data set identified a panel of statistically significantly changing metabolites with VIP > 1 and *p* < 0.05. The second stage of the analysis highlighted the unique benefits of FT-ICR-MS. Instead of a standard database search of metabolite reference MS spectra, Zhang et al. leveraged the high-resolution capabilities of FT-ICR-MS by completing a second analysis of the sample fractions using DI-FT-ICR-MS [25]. The analysis of the isotopic fine structure without chromatographic interference provided the likely elemental compositions of each observed *m*/*z* peak of interest previously determined by HPLC-FT-ICR-MS. The combination of accurate mass, assigned formula, and MS/MS data resulted in metabolite identifications at a much higher confidence (<1 ppm mass error) than is possible with other MS methods. The authors provided specific examples demonstrating how they were able to narrow down results from multiple possible chemical formulas to a single solution. For example, an *m*/*z* peak at 525.37545 was consistent with four possible chemical formulas when only relying on the retention time (RT) and the exact mass from the HPLC-FT-ICR-MS data sets. By including the IFS analysis, only one chemical formula was consistent with all the available data. The *m*/*z* peak at 525.37545 was identified as a lysophosphatidylcholine (LPC (18:0)), with a further confirmation by collision-induced dissociation (CID), yielding a 100% confident peak identification (Figure 2) [25].

High-resolution mass spectrometry (HRMS) provides granular insights into complex and heterogenous metabolomics samples through the use of high mass accuracy and better sensitivity. HRMS is also rapidly evolving to allow for greater sample throughput and increased reproducibility. Zhu et al. (2021) demonstrated the ability to combine flow injection electrospray (FIE) with FT-ICR-MS to provide a continuous flow to the electrospray ionization (ESI) source [29]. FIE is a more robust method than direct injection, with a syringe pump or nano-ESI. Zhu et al. (2021) searched for type 2 diabetes (T2D) biomarkers in genetically identical obese mice. Plasma samples collected from the mice were subjected to a simple methanol extraction to remove interfering proteins and enrich metabolite concentrations. The metabolome samples were then directly injected into the FT-ICR-MS via FIE without LC separation. This method allowed for the rapid simultaneous detection of both polar and non-polar metabolites, with a low percent coefficient of variation (%CV) in peak intensities between multiple replicate injections. Despite the lack of RT and metabolite separation, the high resolution and IFS analysis still allowed for high confidence in the metabolite annotation. Out of 1000 reproducibly detected and annotated metabolites, over 300 metabolites were determined to be statistically distinct between T2D mice and healthy controls. Zhu et al. (2021) highlighted the benefits of HRMS in an incredibly efficient study that provided high-quality data from plasma samples in under 35 min from sample preparation to data acquisition [29]. This rapid analysis allowed for 12 samples to be analyzed in a single hour of instrument time, which is a 4× reduction in total instrument time compared to the ~20 min/sample method that a previous study used [25]. The average LC-MS metabolomics experiment is dependent on many factors, but often takes 45–90 min per sample. Thus, a throughput of 12 samples per hour is a drastic improvement, which has positive implications for the future application of FT-ICR-MS to large-scale metabolomics studies consisting of hundreds to thousands of cohorts.

## 4. FT-ICR-MS in the Search for Non-Human Biomarkers

The search for plant and animal metabolite biomarkers is an equally important area of omics research. For example, a review by Maia et al. (2023) offers some excellent insights into how FT-ICR-MS can be applied to various plant species [30]. The review provides a comprehensive publication list organized by the type of study and the plant species of interest. Plants have high significance for both the environment and human health, and gaining a more complete understanding of their primary and secondary metabolism is an important, if not difficult and complex, pursuit. The number of plant-based metabolites has been estimated to range from 200,000 to upwards of a million compounds, with only a small fraction of these metabolites being detected and identified [31]. In this regard, the application of FT-ICR-MS to various plant metabolomes may assist in generating new plant metabolome databases, since it can detect thousands of compounds in a single cell or cell compartment [30].

The application of FT-ICR-MS to animal metabolomics research, excluding studies on mice and rats as models for human disease, remains limited. Tran et al. (2020) published a review article on the use of metabolomics to study animal diseases [32]. Of the 38 studies on dogs, cats, cows, sheep, horses, pigs, and fish covered in the review, only two used FT-ICR-MS. Both studies focused on flatfish, which are commonly used as a model species in fisheries’ research and as indicators of environmental health in marine environments [33,34,35].

Stentiford et al. (2005) used a homebuilt 9.4T FT-ICR MS to compare the metabolomes from flatfish (*Limanda limanda*) liver tissues with and without the presence of tumors [35]. Only four metabolites were notably altered in flatfish liver tissues with cancerous tumors. The potential chemical formula for these metabolites was derived from accurate mass and IFS analysis. Three of these metabolites were decreased in response to cancer but were not sufficient individually (*p* < 0.10) to distinguish between cancerous and non-cancerous tissue. However, combining the three metabolites into a single biomarker profile consisting of the sum of the three metabolites was a significant discriminatory marker of disease status (*p* < 0.05) [35].

In a follow-up study, Mirbahai et al. (2013) investigated the one-carbon metabolic profile of cancerous flatfish to better understand the underlying cellular mechanisms driving tumorigenesis [34]. Statistically significant (*p* < 0.05) increases were observed in *S*-adenosylhomocysteine, adenosine, and methionine, while choline was found to significantly decrease. It was unclear whether these metabolic changes predisposed the fish to carcinogenesis or were a secondary response to the presence of the tumors. These types of questions remain unanswered not only in flatfish, but also in many other animal species, including humans.

## 5. FT-ICR-MS for Food and Beverage Metabolomics

According to the Food and Beverages E-Commerce Global Market Report 2024, the food and beverages industry is worth about 7–8 trillion USD in 2024 and will reach 9 trillion USD by 2028 (https://www.researchandmarkets.com/reports/5939677/food-beverages-e-commerce-global-market-report, accessed on 1 August 2024). This vast market generates significant research demand, with metabolomics playing a key role in gaining deeper insights into the complex biochemical processes involved in food and beverage production.

Analytical techniques are commonly used to analyze food quality and purity [36]. In recent years, metabolomics has been applied to fingerprinting and categorizing food and beverages by production location, production method, or processing method. Metabolomics is additionally employed to evaluate food and beverage authenticity. Many alcoholic beverages undergo an aging process, and counterfeiting wine and whisky can be a lucrative business due to the high value of certain vintages. Similarly, the inherent value of other food and beverage products is dependent on aging and other characteristics such as locality or origin, brand recognition, species, composition, production method, and rarity. Determining the authenticity of food and alcoholic beverages can be challenging, as fraudulent bottles, labels, corks, and packaging can be reproduced with high accuracy. Therefore, new, advanced analytical methods are needed to classify food and alcohol as authentic or fraudulent, as well as to perform routine quality control and composition analyses. Metabolomics can fingerprint and characterize flavonoid, aromatic, and other critical compounds and is thus well-suited for authenticating food and alcoholic beverages. Whisky, wine, beer, and other spirits are complex biological mixtures. Each step of their production—such as raw material selection, mashing, fermentation, distillation, aging, and bottling—introduces unique chemical compounds that contribute to the overall flavor profile. For example, the region where grapes are grown affects wine, the type of wooden barrel used for aging influences whisky, and the grain used in brewing impacts beer’s flavor. Just as metabolomics is used to distinguish between control and clinical biological samples, it can also identify the chemical compounds at each stage of the manufacturing process. This is crucial for producing high-quality spirits and addressing counterfeiting challenges. To date, FT-ICR-MS has been successfully applied to resolve several of these challenges [37,38,39].

**Figure 3:**
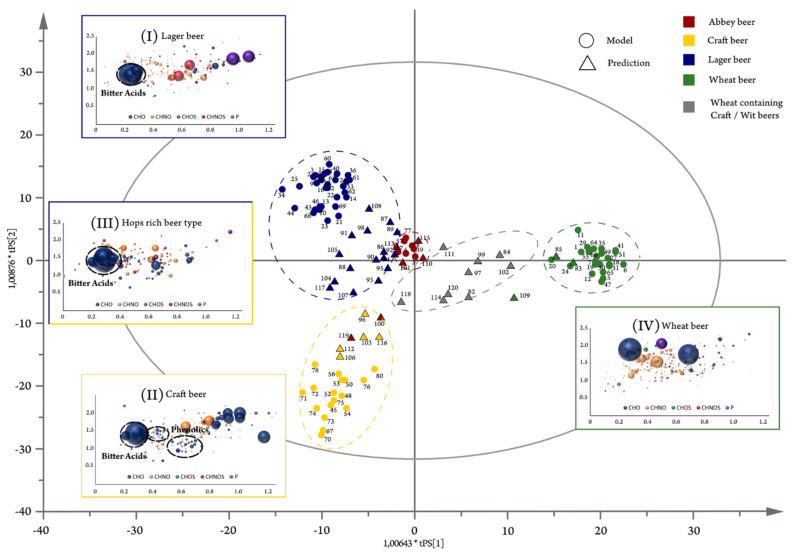
Example of OPLS−DA and van Krevelen plots. The score plot is surrounded by the different observations’ van Krevelen diagrams (lager beers (**I**); craft beers (**II**); rich hopped beer types (**III**); and wheat beers (**IV**)). Samples included in the model calculation are depicted as circles, whereas predicted samples are represented as triangles. Craft and lager beers are summarized as hops-rich beer types to reflect the separation of metabolites in the first component. Color code: CHNO blue; CHNO orange; CHOS green; CHNOS red; P violet; Cl light violet. The bubble size indicates the mean relative intensities. Reproduced with permission from Nature Partner Journals [41].

Kew et al. (2017) used FT-ICR-MS-derived metabolic profiles to determine that the barrel aging process was a primary factor in differentiating whiskeys [37]. Roullier-Gall et al. (2018) expanded upon Kew et al. (2017)’s work by determining which set of compounds that were derived from the cask contributed to these whiskey differences [39]. The whiskey samples were analyzed by direct-injection FT-ICR-MS followed by QToF-MS/MS analysis for structural confirmation. Initial screening provided ~6000 unique *m*/*z* values, with 50% of them having assigned molecular formulas with 0.2 ppm mass error tolerance. However, 64% of the chemical formulas identified did not result in database identification hits. Roullier-Gall et al. (2018) concluded that there were 10 barrel-related metabolite markers, such as syringic acid, caffeic acid, catechin, and epigallocatechin. These metabolites were identified by FT-ICR-MS as distinct markers for the type of barrel used to age the whisky, but were not present in high enough concentrations to be observed or confirmed by LC-MS/MS.

van Krevelen diagrams, which plot the ratio of oxygen to carbon (O/C) vs. the hydrogen to carbon (H/C) ratios of elemental formulas, are often used to visualize and characterize data from natural organic matter [40]. Specific molecular classes often occupy unique regions within the van Krevelen plot and can be used to identify whether an unknown biomolecule is a carbohydrate, lipid, or protein. FT-ICR-MS and IFS analysis are particularly well suited to generating van Krevelen plots, since the high-resolution data offer precise information about the elemental composition. An example of a van Krevelen plot is shown in Figure 3 [41]. Roullier-Gall et al. (2018) used van Krevelen plots to determine which compounds were unique to barrel-aged whisky compared to other barrel-aged alcohols like rum [39]. A higher concentration of compounds containing the elements C, H, and O were identified in the high-alcohol and high-fatty acid regions of the van Krevelen plots. These compounds specifically helped to provide whiskey with its distinct flavor profile [39].

Beer brewing is an intricate process that creates a complex biofluid consisting of a diverse array of chemical compounds. Pieczonka et al. have published several papers that use FT-ICR-MS metabolomics to study beer and its brewing process [41,42,43,44]. Beer metabolic fingerprinting can be completed quickly and accurately with latent structures discriminant analysis (OPLS-DA) statistical models and van Krevelen plots. First, the authors analyzed 85 beers bottled by different countries and highlighted their inherent complexity by identifying the presence of 27 *m*/*z* values within the single nominal mass of 391 [41]. These 85 beers were then differentiated based on unique metabolic profiles influenced by the different grain compositions used during the brewing process, such as wheat, wheat starch, wheat and barley, or barley and hops. An OPLS-DA scores plot shown in Figure 3 demonstrates the separation between these 85 beers based on grain composition, with an overlay of van Krevelen plots highlighting the major molecular classes contributing to these metabolome differences [41]. A univariate statistical analysis suggested that lagers were rich in humulone, cohumulone isomers, and derivatives, which are metabolite markers for hops-rich beers (*p* < 0.001). Other metabolomics analyses from Pieczonka et al. provided insights into the Maillard reaction and the evolution of the beer metabolome throughout the brewing process [42,44], in addition to determining specific metabolite markers for corn and rice beer [43].

Wine authentication has similar aims to beer research, with the interest lying in distinguishing wine by region, vineyard, and vintage, among other factors. A review by Sousa Silva et al. (2024) concisely covers the unique value that FT-ICR-MS can provide to wine metabolomics [45]. Specifically, FT-ICR-MS metabolomics yields clear distinctions between wines based on grapevine selection, chemical composition, and yeast strains in ways that other, less sensitive instrumentation cannot. Another area of interest for wine makers and consumers is the level of sulfites in wine. Sulfites are added to wine to reduce oxidation, but have negative health effects [46]. Another study from Roullier-Gall et al. (2017) demonstrated that wines can be separated based on the concentration of sulfites added to the wine at bottling [47]. The sulfates added at the beginning of the wine-making process have immediate and significant effects on the wine metabolome that continue to change and evolve as the wine ages, creating SO_2_ conjugates that are correlated with the original concentration of the sulfate additive. A multivariate principal component analysis (PCA) displayed clear clustering in the corresponding scores plot based on the amount of sulfites added during bottling. A univariate ANOVA test showed significant (*p* < 0.01) increases in 323 *m*/*z* peak intensities and decreases in 363 *m*/*z* ions. Although less than 15% of the total compounds were annotated, the overall metabolic fingerprint of the wines was easily discriminated with FT-ICR-MS analysis [47].

FT-ICR-MS has been successfully applied to other food and beverage concerns in addition to wines and spirits, such as developing methods to detect the presence of flame retardants in food and vegetable oils [48,49], analysis of the degradation of coconut water [50], monitoring the cocoa bean fermentation process [51], and evaluation of the effects of microplastics in drinking water [52]. Detecting contaminants is often a major goal for HRMS food and beverage analysis, and FT-ICR-MS methods are quickly advancing as an effective choice.

## 6. FT-ICR-MS in Environmental Applications

Environmental research has also benefitted from the application of metabolomics, which has included the detection of toxins in water and soil, the analysis of plant–environment interactions, an examination of food web dynamics, and an evaluation of the impacts of human activities on ecosystems [53]. Unlike clinical metabolomics, which focuses primarily on human health, environmental metabolomics accounts for the influence of numerous plant and animal species, each with unique biological characteristics and requirements, necessitating powerful computational methods to define these environmental relationships [54,55]. Environmental metabolomics must also consider the effects of external factors such as weather conditions, pollutants, pests, and the availability of water and other nutrients that may affect the results [54,55]. The impact of water, soil, and the atmosphere on the study subject are often important research targets because of an intimate interconnection with many fundamental biological processes.

Investigating the amount of dissolved organic matter (DOM) in the environment is key to understanding the global carbon cycle [56]. Traditional environmental research methods that evaluate environmental organic matter, such as chemical oxygen demand or total organic carbon measurements, require large 10–50 mL sample sizes [57,58]. However, an important advantage of employing FT-ICR-MS in an environmental metabolomics project is the significant increase in the amount of data that can be obtained from only 0.5 µL, which is a 1000× reduction in sample volume. Recently, the investigation of the content and concentrations of DOM in water has begun to rely on FT-ICR-MS [11,52,59,60,61,62]. Several recent publications, including a 2023 critical review, have highlighted the beneficial impact of FT-ICR-MS on water analysis [57,59,61,63].

The characterization and cataloging of environmental contaminants and pollutants, such as crude oil, microplastics, and pharmaceuticals, are key research objectives of water research [52,64]. Crude oil is a complex, heterogenous mixture that, among other components, includes water-soluble toxic materials [65,66]. There are additional microscopic oil droplets and insoluble fine particles that are difficult to remove from water [67,68]. Notably, crude oil fractions that are water-soluble or un-removable, designated as water-soluble fractions (WSFs) or water-accommodated fractions (WAFs), respectively, are particularly difficult to characterize. Distinguishing between these two crude oil fractions has been historically difficult and has led to misinterpretations of data and erroneous conclusions [64]. However, Liu et al. (2015) successfully used FT-ICR-MS to characterize the metabolite fingerprint of these water-soluble and un-removable crude oil fractions in order to consistently and accurately distinguish WSF from WAF [64]. By evaluating the ratio of heteroatoms to carbon (NSO:C), FT-ICR-MS identified unique crude oil-derived polar compounds present in WAF and WSF. A major distinction between crude oil and the WSF and WAF fractions was the low heteroatom counts in the crude oil compounds. Conversely, the dominant molecules in the WSF and WAF fractions contained at least three heteroatoms. Additionally, NSO:C values were significantly higher in WSF than in WAF. The proper identification of a WAF or WSF marine pollution condition has a significant impact on understanding the outcoming (i.e., organismal toxicity) or how to implement a response. This was not feasible when WAF and WSF were previously interchangeably and indistinguishable. Overall, FT-ICR-MS is well suited for water metabolome analysis and evaluating water for purity, or for investigating the nature of the contaminants.

Soil serves as the foundation for plant growth, supports various life forms, and acts as a natural water filter, making it a major target for environmental metabolomics research [69,70,71]. Soil remediation is the process of removing, reducing, or neutralizing soil pollutants and contaminants to make it safe for humans, animals, and plants to interact with [72]. Remediation strategies for contaminated soil include physically removing and disposing of the soil, washing the soil, adding microorganisms, or heating the soil to facilitate chemical decomposition or breakdown of the pollutants [73]. Chemical oxidation is a remediation process that involves the addition of oxidants like hydrogen peroxide or sodium persulfate (PDS) to the soil to oxidize and convert the pollutants into inert material. The Fenton reaction uses transition metals like Fe, Cu, and Zn to catalyze hydrogen peroxide to transform into hydroxyl radicals, which can then react with contaminants in soil for conversion into inert compounds (generally CO_2_ or H_2_O, depending on the type of contaminant) [74]. The oxidation process is affected by the presence and composition of the soil organic matter (SOM), which has vital effects on soil health and bioavailability and can positively or negatively influence soil pollutant degradation [70]. Little is known about the mechanism by which SOM affects the chemical oxidation remediation process.

Geng et al. (2023) conducted a targeted soil metabolomics study seeking to identify the molecular composition of SOM [63]. Diesel-contaminated soil was collected and analyzed via FT-ICR-MS to characterize the SOM content before and after chemical remediation through the addition of PDS or H_2_O_2_. The goal of the project was to understand how the SOM transforms during the remediation process. The two primary analysis methods were Kendrick mass defect (KMD) analysis and van Krevelen plots (previously described in Section 5), which are positively influenced by higher MS resolution. KMD analysis is similar to van Krevelen plots, as it provides a means to visualize and group molecules by class or composition. The concept is based on the Kendrick mass scale, which normalizes the mass of a molecule to a common fragment (e.g., CH_2_ group) [75,76,77]. In a KMD plot, the Kendrick mass is plotted on the *X* axis and the Kendrick mass defect is plotted on the *Y* axis. The resulting plot allows for the visualization of a homologous series of compounds, which appear as horizontal lines. This method is especially useful for identifying patterns and structural similarities within complex mixtures, aiding in the elucidation of molecular compositions and the identification of compound classes. Briefly, to calculate the Kendrick mass, the mass (Da) of each detected ion is multiplied by a conversion factor. The conversion factor can vary, although it is typically the nominal mass of CH_2_ divided by the exact mass of CH_2_ (e.g., 14.00000/14.01565). The Kendrick mass defect is the difference between the nominal Kendrick mass and the Kendrick exact mass. As an example, alanine (C_3_H_5_NO) has an exact mass of 71.037114 Da, a Kendrick mass of 70.95779 Da, and a Kendrick mass defect of 0.90422 Da.

Overall, FT-ICR-MS metabolomics clearly demonstrated that a PDS treatment of soil had a positive effect on soil remediation through an increased degradation of a wider range of alkane chain lengths with less carbon dioxide production [63]. Additionally, SOM content transformed into a higher level of unsaturated and carboxyl-containing compounds during PDS treatment, which are positive indicators of soil health. H_2_O_2_ soil treatment produced the opposite effect [63].

Finally, the atmosphere, which regulates climate and weather patterns and helps to disperse pollutants, is another area of environmental research interest. Cloud water refers to the tiny droplets of liquid water that form clouds in the Earth’s atmosphere. Cloud water contains microorganisms that can greatly vary cloud oxidative capacity and atmospheric processes [78]. By incubating cloud water at different temperatures, Bianco et al. (2019) were able to study the endogenous metabolism of cloud water microorganisms [79]. FT-ICR-MS was used to assign molecular formulas to the metabolites formed and consumed by the microorganisms under different experimental conditions. Temperature was observed to have the largest effect on metabolism, where cloud microorganisms degraded more compounds at 5 °C (1716 metabolites consumed and 173 metabolites produced) compared to the 1094 compounds consumed at 15 °C. More compounds were biosynthesized at 15 °C (266) compared to the 173 compounds produced at 5 °C. It was interesting to note that the presence of light had no significant effect on metabolism. Additionally, specific trends were identified for several groups of biomolecules. For example, more saccharides were consumed at lower temperatures, while carboxylic-rich alicyclic molecules were produced at higher temperatures. Carboxylic-rich alicyclic molecules were highly affected by microbial activity and, thus, were consumed to maintain microbial metabolism. Interestingly, higher-molecular-weight compounds were produced with an increase in microbial activity, which can alter droplet surface tension and affect the cloud’s ability to transfer between the gas and aqueous phase [79].

FT-ICR-MS has proven to be highly successful across a wide range of environmental applications. Its ability to resolve IFS provides valuable insights into previously unexplored scientific questions. By enabling precise molecular formula determination, FT-ICR-MS allows for a comprehensive analysis of various environmental samples. Notable applications include analyzing trends between saturated and unsaturated compounds and comparing ratios of carbon to other heteroatoms (NOSP). Additionally, FT-ICR-MS facilitates highly accurate van Krevelen and KMD diagrams. van Krevelen diagrams help to identify the degree of oxidation and unsaturation of organic compounds, while KMD diagrams aid in recognizing homologous series and structural motifs within samples [75,80]. FT-ICR-MS is enhancing our understanding of environmental processes, the effects of pollution, and is providing valuable insights to improve environmental monitoring and remediation.

## 7. FT-ICR-MS High Resolution Imaging

Matrix-assisted laser desorption ionization (MALDI) is a widely used technique in mass spectrometry for ionizing samples [81]. The MALDI process begins by mixing the sample of interest with a volatile organic solvent and then applying it to a stainless steel MALDI plate. The small organic acid serves as a matrix and is added at a ratio of 1000:1 (matrix:analyte) to the sample. The prepared sample is then introduced into the mass spectrometer vacuum chamber. A laser is fired at a specific X, Y coordinate along the sample surface, transferring energy from the matrix to the analyte and resulting in sample ablation and ionization. The resulting laser-induced “hole” in the sample surface may range from 10 to 200 mm. Replicate analysis typically requires the use of a different location on the sample surface, where it is rare to reanalyze the same location or the newly created surface hole. MALDI is particularly useful for analyzing large-molecular-weight samples because it generally produces singly charged ions, simplifying data interpretation [82].

MALDI imaging is a technique that was introduced in the mid-1990s and has been rapidly adopted due to recent advances in resolution with FT-MS instruments [83]. The process of MALDI imaging involves spraying a matrix onto a glass-slide-mounted tissue slice and then rastering (i.e., changing the X, Y coordinate) the laser across the tissue to generate hundreds or thousands of mass spectra. The benefit of MALDI imaging compared to other traditional metabolomics methods is the retention of spatial information. Each *m*/*z* peak from each mass spectrum can be correlated to a precise location on the tissue sample. Traditionally, LC-MS metabolomics experiments rely on the solvent extraction of a homogenized tissue sample to collect the entire metabolome, which loses all spatial information. The tissue is also destroyed in the extraction process. Nevertheless, MALDI imaging experiments can be cumbersome on FT-MS instruments and can take hours or days to complete. Small tissue slices at relatively lower (~200 µm) spatial and MS resolutions will produce smaller data files than large tissue samples at high (~20 µm) spatial and MS resolutions. The data files can range from 10 to 100 gigabytes in size depending on the experimental parameters. Please see previously published reviews on MALDI imaging for practical experimental and processing details [84,85,86]. Instead, a selection of unique and intriguing applications of MALDI-FT-ICR-MS imaging to metabolomics will be provided to explore how its high spatial and mass resolution offer unmatched insights into the metabolic processes.

**Figure 4:**
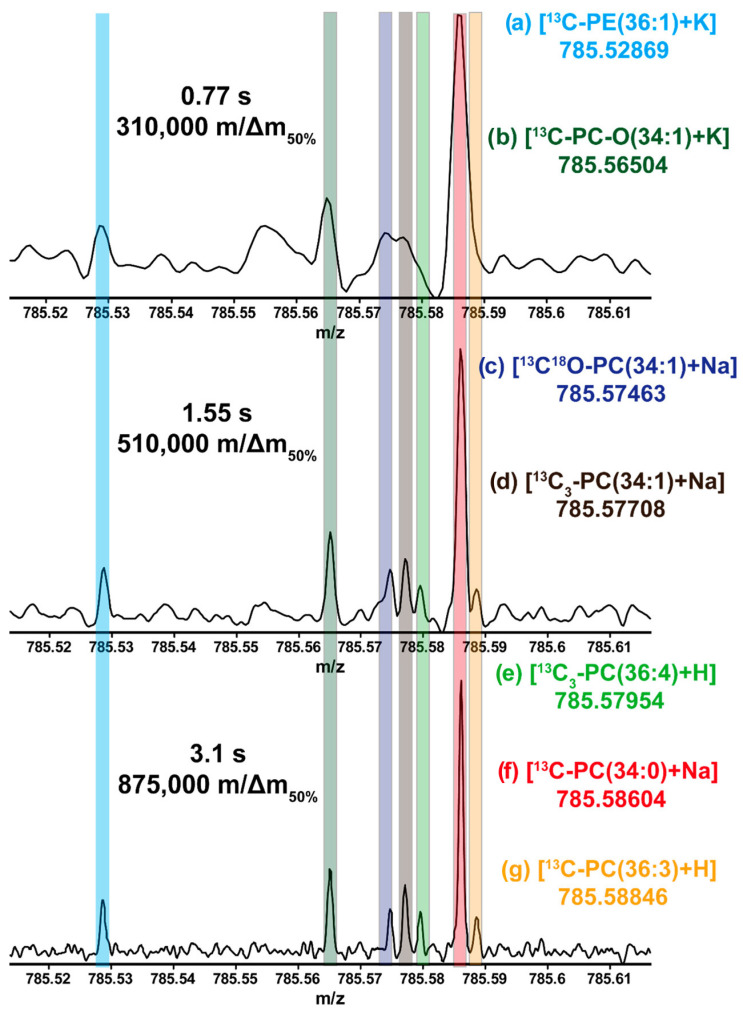
Example of HR-MS resolving power increasing with transient length. Mass resolution and sensitivity improve with longer transient lengths. Within a 100 mDa mass range, seven different peaks are detected which belong to six different lipid species. Of these, five are unresolved at 0.77 s. While distinguishable at 1.55 s, all seven peaks are fully resolved only at 3.1 s transient. These seven peaks correspond to the isotopologues of the monoisotopic species, typically the ^13^C ion, as in (a), (b), (f), and (g). Other species are also present, corresponding to the ^13^C_3_ isotopologue, as in (d) and (e). The ^18^O^13^C isotopologue of [PC(34:1) + Na]^+^ is also resolved (c) from the ^13^C_3_ isotopologue of the same parent species. Reproduced with permission from the American Chemical Society [17].

As previously mentioned, and as shown in Eqn. 1, FT-ICR-MS resolution increases linearly with magnetic field strength. Currently, a 21 T magnet is the highest field FT-ICR-MS available. Bowman et al. (2020) used the 21 T FT-ICR-MS at the National High Magnetic Field Laboratory ICR User Facility (Tallahassee, FL, USA) to image rat brain tissues [17]. A HRMS lipidomics analysis revealed previously hidden features within the narrow 700 to 900 *m*/*z* range, which lower field instruments cannot detect. A unique set of seven lipids within a 0.22 *m*/*z* window and a 13 to 50 ppb mass error was detected, leading to an exceptionally high confidence in the lipid assignments (Figure 4). A total of 2643 (positive mode) and 1927 (negative mode) *m*/*z* peaks were above the 6σ noise threshold in the mass spectra. In total, 2102 *m*/*z* peaks (80%) from the positive mode analysis and 1400 *m*/*z* peaks (73%) from the negative mode analysis were tentatively identified as lipids or lipid isotopologues. Most of these lipid identifications had mass errors of <100 ppb. The *m*/*z* peak intensity ratios were similar in both the positive and negative modes. In the positive mode, the ratios of [M + K], [M + Na], and [M + H] adducts were consistent. This exceptionally high percentage of confident peak annotation is unique to FT-ICR-MS. Thus, FT-ICR-MS MALDI imaging is poised to become an increasingly vital tool in advancing metabolomics research as progress in field strength and ICR cell ion capacity/suppression continues.

An improvement in FT-ICR-MS imaging can be achieved by coupling a standard ionization method like MALDI with desorption electrospray ionization (DESI) to gain a multi-modal imaging technique that can analyze a broader range of biomolecular classes. Zemaitis et al. (2021) were the first to complete a multimodal experiment with a single FT-ICR-MS instrument [87]. A comparison of the DESI and MALDI results indicated that the lipid fingerprint for each ionization method was unique. Accordingly, combining the data sets led to expanded and comprehensive coverage of the lipidome. A total of 288 lipid peaks were annotated from DESI analysis, and 404 from MALDI. The FT-ICR-MS platform was able to resolve nearly all isobaric species. Notably, this experiment was completed with no modifications or external software to the system, which streamlines the approach compared to previous workflows.

MALDI and MALDI imaging have revolutionized mass spectrometry, especially when integrated with high-resolution techniques like FT-ICR-MS. This combination enhances molecular exploration, offering precise spatial identification and characterization of biomolecules, including those in low abundance. MALDI imaging reveals detailed biomolecular distributions within tissues, which advances our understanding of biological processes.

## 8. Practical Considerations for Implementing FT-ICR-MS

### 8.1. FT-ICR-MS Locations and Sample Submission Costs 

As demonstrated in the previous sections, FT-ICR-MS offers unique capabilities that make it highly suitable for omics research. However, a primary drawback is cost. FT-ICR-MS instruments range in price from USD 500,000 to USD 5 million depending on the manufacturer and magnet strength (1.2 to 21 T). The instrument’s expense can be mitigated or avoided by submitting samples to national mass spectrometry facilities that operate FT-ICR-MS instruments. Hourly or per-sample facility fees can still be costly. Furthermore, mass spectrometry facilities operate differently and may provide a diverse range of services; some facilities may offer full collaborative assistance, while others may only provide routine data acquisition services.

Due to prohibitive costs, FT-ICR-MS instruments are limited worldwide. In the United States, there are only a few dozen FT-ICR-MS instruments, but many have limited access to specific research groups and are not available for general use. The National High Magnetic Field Laboratory (Tallahassee, FL, USA) is a premier location for magnetic resonance mass spectrometry that houses the world’s highest field FT-ICR-MS at 21 T, which is available for commercial use. Table 2 is a non-comprehensive list of the FT-ICR-MS instruments available for general use located at various facilities throughout the US and the United Kingdom (UK). For the most up-to-date facility fees and pricing information, please consult the individual facility’s website.

### 8.2. FT-ICR-MS Sample Preparation Concerns

Before samples are ready for metabolomics analysis, complex and proper sample preparation is essential to ensure accurate and reliable results. Please see previously published method papers for a detail description of experimental protocols [88,89]. Briefly, the metabolomics sample preparation process typically involves removing matrix background, proteins, and other biomolecules; enriching the metabolites of interest; and maintaining uniform conditions across all samples. Important considerations to circumvent biologically irrelevant bias include rapid, cold, and randomized sample processing; avoiding long storage times (even at −80 °C); excluding sample stability additives (i.e., no EDTA, glycerol, or other compounds); controlling sample pH and osmolality (i.e., add buffers); minimizing the number of individuals processing samples (i.e., one person per task); and including the use of standards, internal references, blanks, and quality control samples. Meticulous and rigorous sample preparation protocols are particularly crucial for achieving precise and accurate FT-ICR-MS analysis and MALDI imaging due to the platform’s increased sensitivity.

### 8.3. Sample Purity and Concentration

The higher sensitivity of FT-ICR-MS necessitates careful consideration of sample concentration and purity. Contaminants introduced during sample preparation, handling, and storage can overwhelm the signals of the analytes of interest, leading to erroneous conclusions. Additionally, FT-ICR-MS can detect compounds present in femtomolar concentrations which are magnitudes lower than what is required for other MS platforms. Overloading the ICR cell with too many ions can result in ion suppression effects, broader peaks, and an overall lower quality of data [90,91].

### 8.4. When Possible, Use Fresh Frozen Tissues for Mass Spectrometry Imaging

Histopathology is the study of tissue disease at a microscopic level and often involves staining tissue samples to understand cellular architecture, composition, and abnormalities or to stabilize the sample for analysis. For example, formalin-fixed, paraffin-embedded (FFPE) tissues are commonly used because the cells are resistant to decay and have a higher quality and long-term structural integrity [92]. However, embedded or cryopreserved tissues present a challenge for mass spectrometry imaging, since the fixing agents can suppress the analytes of interest. These fixing agents can be removed through solvent washes and soaking, but the removal process can degrade the tissue and potentially remove analytes of interest [93].

Fresh frozen tissue (FF) is the gold-standard sample for MALDI imaging. Unfortunately, sometimes, an FFPE tissue is unavoidable, particularly in retrospective studies. Various methods have been optimized to remove FFPE with minimal sample degradation and maximum data retrieval. For example, Buck et al. (2015) demonstrated that biomedically relevant information remains in FFPE tissue samples after removing the paraffin with solvents like xylene or isopropanol [93]. Please see a published tutorial that details the procedures for removing FFPE from tissue sections for MALDI imaging, including on-tissue derivatization and digestion [94,95]. Despite these advancements, fresh frozen tissue remains the preferred choice due to concerns about metabolite loss during the FFPE removal processes [93].

These broad points are intended to guide future project planning and decision making regarding the implementation of FT-ICR-MS in a metabolomics study. Location, cost, and sample preparation considerations are critical aspects of all analytical analyses, but the unparalleled benefits of FT-ICR-MS often justify the extra time and effort. The high resolution and spatial accuracy provided by FT-ICR-MS uniquely enables detailed imaging of biomolecular distributions within tissues, making it a valuable tool for mass spectrometry imaging. Through careful consideration of these factors, researchers can maximize the potential of FT-ICR-MS to yield insightful and impactful spatial metabolomics data.

## 9. Conclusions

FT-ICR-MS is a valuable tool for metabolomics research due to its exceptional resolution and mass accuracy. Its application in human clinical biomarker research has significantly advanced our understandings of disease mechanisms while also identifying potential therapeutic targets and disease biomarkers. FT-ICR-MS also plays a crucial role in plant, animal, food, beverage, and environmental scientific endeavors. In this regard, numerous metabolomics research projects have benefited from the ability of FT-ICR-MS to precisely profile complex mixtures, to detect low abundances of metabolites and to identify environmental pollutants, among other capabilities. FT-ICR-MS imaging provides both spatial resolution and accurate metabolite annotation within a range of tissue types. FT-ICR-MS can distinguish between multiple isobaric species, provide detailed insights into narrow *m*/*z* windows, and simultaneously identify thousands of metabolites across tissues with high mass accuracy.

Despite its many advantages, implementing FT-ICR-MS in research poses challenges due to its cost, complexity, and limited availability. Obtaining reproducible data requires standardized and optimized protocols with special considerations for sample preparation, particularly for imaging studies. These challenges can be overcome through a collaboration between individual investigators and national mass spectrometry facilities equipped with FT-ICR-MS instruments and employing expert operators and technicians. These specialists can help guide projects to successful outcomes much faster and more efficiently than can be accomplished by inexperienced and novice investigators. Additionally, it is important for FT-ICR-MS and other metabolomics studies to develop and adopt standardized protocols for sample preparation, data collection, and analysis. It is difficult to obtain high-quality and reproducible data without these properly vetted guidelines. Several organizations are currently engaged in the establishment of best practices for metabolomics, but the metabolomics community has yet to widely adopt these protocols [96,97,98,99,100,101]. In summary, FT-ICR-MS offers cutting-edge capabilities for metabolomics research, making it an indispensable platform for comprehensive metabolic analysis across various scientific fields. The continued development of FT-ICR-MS and the diversity of applications hold great promise for enhancing our understanding of complex metabolic processes, contributing to advancements in human and animal health, agriculture, food science, and environmental studies.

## Figures and Tables

**Table 1:** Figures of merit for various MS analyzers.

Analyzer	Accuracy	Resolution	*m*/*z* Range	Scan Speed	General Comments
Magnetic Sector	<5 ppm	30,000	10,000	1 s	Large footprint
Quadrupole	100 ppm	4000	4000	1 s	Low cost
Ion Trap	100 ppm	4000	1000	1 s	Low cost, well suited for MS^n^
Time of Flight (ToF)	200 ppm	8000	>300,000	ms	Low cost
ToF Reflectron	10 ppm	15,000	10,000	ms	Good accuracy, good resolution
Quadrupole-TOF	10 ppm	10,000	10,000	1 s	High sensitivity and accuracy when used for MS^2^
FT-ICR-MS	100 ppb	10^6–7^	10,000	1–10 s	Expensive, large footprint but has highest accuracy and resolving power
Orbitrap	1 ppm	10^5–6^	10,000	1 s	Faster scan speeds allow for easier combination with LC systems

**Table 2:** Partial list of FT-ICR-MS facilities in the US and the UK.

Facility	Location
National High Magnetic Field Laboratory (MagLab) at Florida State University	Tallahassee, FL
The Ohio State University	Columbus, OH
University of Alabama at Birmingham	Birmingham, AL
University of Nebraska-Lincoln	Lincoln, NE
University of Wisconsin-Madison	Madison, WI
Florida International University	Miami, FL
Old Dominion University	Norfolk, VA
University of Maryland School of Pharmacy	Baltimore, MD
Weill Cornell Medicine	New York, NY
Woods Hole Oceanographic Institution	Woods Hole, MA
European Network of FT-ICR-MS Centers	Various (www.eu-fticr-ms.eu, accessed on 1 August 2024)
University of Birmingham (UK)	Birmingham, UK

## Supplementary Materials

The following supporting information can be downloaded at: https://www.mdpi.com/article/10.3390/biomedicines12081786/s1, Table S1: Summary of Metabolomics Projects Discussed in the Review Paper.

## List of Abbreviations

AD	Alzheimer’s disease
ANOVA	Analysis of variation
CID	Collision induced dissociation
CV	Coefficient of variation
DESI	Desorption electrospray ionization
DI-FT-ICR-MS	Direct injection FT-ICR-MS
DOM	Dissolved organic matter
ESI	Electrospray ionization
FF	Fresh frozen
FFPE	Formalin-fixed, paraffin-embedded
FID	Free induction decay
FIE	Flow injection electrospray
FT-ICR-MS	Fourier transform ion cyclotron resonance mass spectrometry
H/C	Hydrogen to carbon ratio
HMDB	Human metabolome database
HPLC	High performance liquid chromatography
HRMS	High resolution mass spectrometry
ICR	Ion cyclotron resonance
IFS	Isotopic fine structure
KMD	Kendrick mass defect
LC-MS	Liquid chromatography mass spectrometry
LPC	Lysophosphatidyl choline
MALDI	Matrix assisted laser desorption ionization
MS	Mass spectrometry
NMR	Nuclear magnetic resonance
NSO:C	Ratio of heteroatoms to carbon
O/C	Oxygen to carbon ratio
OPLS-DA	Orthogonal projections to latent structures discriminant analysis
PDS	Sodium persulfate
ppb	Parts per billion
QToF	Quadrupole time-of-flight
RCE	*Rhodiola crenulate*
RT	Retention time
SOM	Soil organic matter
T	Tesla
T2D	Type 2 diabetes
UK	United Kingdom
WAF	Water accommodated fraction
WSF	Water soluble fraction
