# Supplementary material for: Fourier Transform Ion Cyclotron Resonance Mass Spectrometry Applications for Metabolomics
Source: Biomedicines. 2024 Aug 6;12(8):1786. doi: 10.3390/biomedicines12081786 (PMC11351437; doi:10.3390/biomedicines12081786)
Supplement: Supplementary file 1 [file biomedicines-12-01786-s001.zip › biomedicines-3123258-supplementary.pdf]

Supplemental Information

Fourier Transform Ion Cyclotron Resonance Mass Spectrometry Applications for Metabolomics

Darcy Cochran [1,2] and Robert Powers [1,2,*]

[1] *Department of Chemistry, University of Nebraska-Lincoln, Lincoln, 777 Hamilton Hall, Licoln, NE 68588-0304, USA; darcy.lin@huskers.unl.edu*

[2] *Nebraska Center for Integrated Biomolecular Communication, University of Nebraska-Lincoln, Lincoln, NE 68588-0304, USA*

*Correspondence: rpowers3@unl.edu; Tel.: +1-(402)-472-3039; Fax: +1-(402)-472-9402

Keywords: mass spectrometry; metabolomics; FT-ICR-MS; imaging

Table S1: Summary of Metabolomics Projects Discussed in the Review Paper.

Topic	Author	Year	Title	DOI
Human Biomarkers	Zhang, X *et al.*	2019	A metabolomic study based on accurate mass and isotopic fine structures by dual mode combined-FT-ICR-MS to explore the effects of Rhodiola crenulata extract on Alzheimer disease in rats.	10.1016/j.jpba.2019.01.021
	Zhu, Y *et al.*	2021	Ultrahigh-Resolution Mass Spectrometry-Based Platform for Plasma Metabolomics Applied to Type 2 Diabetes Research	10.1021/acs.jproteome.0c00510
Non-Human Biomarkers	Maia, M. *et al.*	2023	FT-ICR-MS-based metabolomics: A deep dive into plant metabolism	10.1002/mas.21731
	Tran, H. *et al.*	2020	Metabolomics in the study of spontaneous animal diseases	10.1177/1040638720948505
	Mirbahai, L. *et al.*	2013	Disruption of DNA methylation via S-adenosylhomocysteine is a key process in high incidence liver carcinogenesis in fish	10.1021/pr400195u
	Stentiford, G. D. *et al.*	2005	Liver tumors in wild flatfish: a histopathological, proteomic, and metabolomic study	10.1089/omi.2005.9.281
Food and Beverage	Kew, W. *et al.*	2017	Chemical Diversity and Complexity of Scotch Whisky as Revealed by High-Resolution Mass Spectrometry.	10.1007/s13361-016-1513-y
	Kew, W. *et al.*	2018	Complementary Ionization Techniques for the Analysis of Scotch Whisky by High Resolution Mass Spectrometry.	10.1021/acs.analchem.8b01446
	Roullier-Gall, C. *et al.*	2018	Usage of FT-ICR-MS Metabolomics for Characterizing the Chemical Signatures of Barrel-Aged Whisky.	10.3389/fchem.2018.00029
	Pieczonka, S. A. *et al.*	2021	Hidden in its color: A molecular-level analysis of the beer's Maillard reaction network.	10.1016/j.foodchem.2021.130112
	Pieczonka, S. A. *et al.*	2020	Decomposing the molecular complexity of brewing.	10.1038/s41538-020-00070-3
	Pieczonka, S. A. *et al.*	2021	On the Trail of the German Purity Law: Distinguishing the Metabolic Signatures of Wheat, Corn and Rice in Beer.	10.3389/fchem.2021.715372
	Pieczonka, S. A *et al.*	2023	FT-ICR-MS reveals the molecular imprints of the brewing process.	10.3389/fnut.2023.1243503
	Sousa Silva, M. *et al.*	2024	From the grapevine to the glass: A wine metabolomics tale by FT-ICR-MS.	10.1002/jms.5019
	Roullier-Gall, C. *et al.*	2017	Sulfites and the wine metabolome.	10.1016/j.foodchem.2017.05.039
	Zacs, D. *et al.*	2019	Evaluation of analytical performance of gas chromatography coupled with atmospheric pressure chemical ionization Fourier transform ion cyclotron resonance mass spectrometry (GC-APCI-FT-ICR-MS) in the target and non-targeted analysis of brominated and chlorinated flame retardants in food.	10.1016/j.chemosphere.2019.03.047
	Wu, Z. *et al.*	2004	Characterization of Vegetable Oils: Detailed Compositional Fingerprints Derived from Electrospray Ionization Fourier Transform Ion Cyclotron Resonance Mass Spectrometry	10.1021/jf049596q
	Costa, H. B. *et al.*	2015	Monitoring the physicochemical degradation of coconut water using ESI-FT-ICR MS.	10.1016/j.foodchem.2014.10.154
	Kuhnert, N. *et al.*	2020	Investigating time dependent cocoa bean fermentation by ESI-FT-ICR mass spectrometry	10.1016/j.foodres.2020.109209
	Lee, Y. K. *et al.*	2023	Effects of organic additives on spectroscopic and molecular-level features of photo-induced dissolved organic matter from microplastics.	10.1016/j.watres.2023.120272

Environmental Applications	Gan, S. *et al.*	2021	A Novel Method for Unraveling the Black Box of Dissolved Organic Matter in Soils by FT-ICR-MS Coupled with Induction-Based Nanospray Ionization.	10.1021/acs.estlett.1c00095
	Bercovici, S. K. *et al.*	2022	The detection of bacterial exometabolites in marine dissolved organic matter through ultrahigh-resolution mass spectrometry	10.1002/lom3.10491
	Ruan, M. *et al.*	2023	Molecular-level exploration of properties of dissolved organic matter in natural and engineered water systems: A critical review of FTICR-MS application	10.1080/10643389.2022.2157167
	Xu, W. *et al.*	2020	Using ESI FT-ICR MS to Characterize Dissolved Organic Matter in Salt Lakes with Different Salinity	10.1021/acs.est.0c01681
	Geng, W. *et al.*	2023	Unveiling Molecular Transformations of Soil Organic Matter after Remediation by Chemical Oxidation Based on ESI-FT-ICR-MS analysis	10.1021/acsestengg.2c00423
	Liu, Y. *et al.*	2015	Chemical Composition and Potential Environmental Impacts of Water-Soluble Polar Crude Oil Components Inferred from ESI FT-ICR MS	10.1371/journal.pone.0136376
	Bianco, A. *et al.*	2019	Effect of endogenous microbiota on the molecular composition of cloud water: a study by Fourier-transform ion cyclotron resonance mass spectrometry (FT-ICR MS).	10.1038/s41598-019-44149-8
MS Imaging	Bowman, A. P. *et al.*	2020	Ultra-High Mass Resolving Power, Mass Accuracy, and Dynamic Range MALDI Mass Spectrometry Imaging by 21-T FT-ICR MS	10.1021/acs.analchem.9b04768
	Zemaitis, K. J. *et al.*	2021	Streamlined Multimodal DESI and MALDI Mass Spectrometry Imaging on a Singular Dual-Source FT-ICR Mass Spectrometer.	10.3390/metabo11040253
